# Alterations of monoamine neurotransmitters, HPA-axis hormones, and inflammation cytokines in reserpine-induced hyperalgesia and depression comorbidity rat model

**DOI:** 10.1186/s12888-022-04065-0

**Published:** 2022-06-22

**Authors:** Jingjie Zhao, Wei Shi, Yujia Lu, Xuesong Gao, Anna Wang, Shan Zhang, Yi Du, Yongzhi Wang, Li Li

**Affiliations:** 1 Department of Traditional Chinese Medicine, Beijing Friendship Hospital, Capital Medical University, No. 95 Yong-an Road, Beijing, 100050 China; 2 Department of Integrated Traditional and Western Medicine, Capital Medical University, Beijing, 100050 China; 3 Beijing Pediatric Research Institute, Beijing Children’s Hospital, Capital Medical University, National Center for Children’s Health, Beijing, 100045 China

**Keywords:** Monoamine neurotransmitters, HPA-axis hormones, Inflammation cytokines, Pain and depression comorbidity

## Abstract

**Background:**

Pain and depression often occur simultaneously, but the mechanism of this condition is still unclear.

**Methods:**

The aim of this study was to examine the alterations of monoamine neurotransmitters, hypothalamic–pituitary–adrenal (HPA) axis hormones, and inflammation cytokines in hyperalgesia and depression comorbidities. The reserpine-induced “Sprague Dawley” (SD) rat models were used, and the concentrations of monoamine neurotransmitters serotonin (5-HT), norepinephrine (NE), dopamine (DA), and their metabolic products 5-hydroxyindoleacetic acid (5-HIAA), Homovanillic acid (HVA), 3,4-Dihydroxyphenylacetic acid (DOPAC) in raphe nucleus region were tested by High Performance Liquid Chromatography (HPLC). Serum levels of Adrenocorticotropic Hormone (ACTH), Cortisol (CORT), and inflammatory cytokines interleukin (IL)-1β, IL-6, tumor necrosis factor (TNF)-α, interferon (IFN)-γ, IL-4, IL-10 were assessed by enzyme linked immunosorbent assay.

**Results:**

Repeated reserpine injection induced hyperalgesia and depressive behaviors with decreased sucrose preference and horizontal movement distance, and increased immobility time in forced swimming test. The concentrations of 5-HT and NE in raphe nucleus, and ACTH and CORT in serum were elevated in the model group. And the model group showed increases in serum IL-1β and IL-6, and decrease in serum IL-10.

**Conclusion:**

More research in these areas is needed to understand the pathogenesis of the disease, so as to find more and better therapeutic targets.

## Background

Pain and depression often occur simultaneously [1]. Epidemiologic studies have shown that pain is a risk factor of depression [2, 3], and depression can increase the risk of pain [2, 4]. The two conditions can often exacerbate each other, resulting in a longer duration of symptoms and worse prognosis, which in turn leads to greater financial costs to the patients and to our society [5, 6]. Although it has long been recognized that pain and depression are often associated with each other, and the research on pain and depression comorbidity has increased significantly in recent years [7], the mechanism of pain and depression comorbidity is still unclear.

Studies dating back to the nineteenth century suggested that changes in neurotransmitters are common in patients with pain [8] and depression [9]. Another potential involvement of the two diseases maybe the hypothalamic-pituitary-adrenal (HPA) axis, as dysfunction of the HPA axis were found in both pain [10] and depression [11]. Recent researches pointed out that neuroinflammatory alterations may also play a role in the pathophysiology of pain [12] and depression [13]. But all of these studies have focused on just one aspect. It has been unknown whether there are changes in all of these aspects in pain and depression comorbidity, or whether there are differences between individuals.

Several modeling methods [14] are used for studying the mechanisms of pain-depression dyad, including nerve injury [15], social stress [16], stress load [17], monosodium iodoacetate [18] and administration of reserpine [19]. Reserpine is a monoamine depletory. Repeated reserpine treatment can induce the phenotype of pain and depression, making it an ideal model for studying pain-depression comorbidity.

In the present study, we assessed the alterations in serum levels of HPA axis hormones and inflammatory cytokines, and measured the concentrations of monoamine neurotransmitters and their metabolic products in the raphe nuclei region based on a reserpine-induced rat model of hyperalgesia -depression comorbidity to better illustrate the underlying mechanisms of this disease.

## Methods

### Animals

**Fig. 1:**
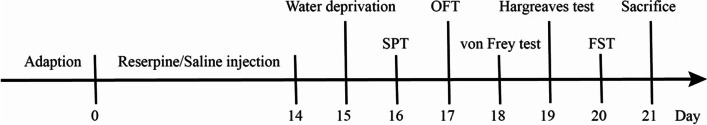
Time schedule of the whole experimental design

Eight-week-old male “Sprague Dawley” (SD) rats weighing 180–200 g were purchased from Beijing Vital River Laboratory Animal Technology Co., Ltd. The rats were maintained in a pathogen-free, temperature-controlled environment with a 12-h light/dark cycle. The animal studies were performed in compliance with the ethical guidelines for animal studies and were approved by the Institutional Animal Care and Ethics Committee of Beijing Friendship Hospital (No. 19–1006). After 1 week of habituation, 20 rats with similar horizontal movement distance in the open field test were selected and randomly divided into model group and control group, with 10 rats in each group. Rats in the model group were intraperitoneally injected reserpine at 0.3 mg/kg body weight every morning for 14 days. Rats in the control group were intraperitoneally injected with normal saline at the same dose at the same time for 14 days. Reserpine injection solution were purchased from Jin Yao Pharmaceutical Company (Tianjin, China). Behavioral tests were performed in the following sequence: sucrose preference test (SPT), open field test (OFT), mechanical hyperalgesia (von Frey test), thermal hyperalgesia (Hargreaves test) and forced swim test (FST). All behavioral tests were performed with a 24-hour interval. The body weight of rats was weighed and recorded every day since the injection began. Blood and the raphe nucleus tissue samples were collected after the final behavioral test. Time schedule of the whole experimental design was shown in Fig. 1.

### Behavioral tests

#### Sucrose preference test (SPT)

SPT was performed as we described earlier [20]. Rats were first given a bottle of 2% sucrose solution and a bottle of pure water for 24 hours, and then the sucrose bottle and the water bottle were swapped for another 24 hours. After the adaptation was completed, the rats were allowed free access to a bottle of 2% sucrose solution and a bottle of pure water for free drinking after 16 h of water deprivation. Intake of the sucrose solution and pure water was recorded for 24 h. Sucrose preference, which is considered as an indicator of anhedonia, was defined as the ratio of sucrose intake to the total intake of both sucrose solution and pure water.

#### Open field test (OFT)

The OFT was performed in a 100 × 100 × 40 cm^3^ black wall box with the SMART (v3.0.03) video-tracking system software (Panlab Harvard Apparatus, Barcelona, Spain). The arena was divided into 4 equal squares, and the rats were individually placed in the center of each square and allowed to move freely for 5 minutes. The movement of the rat was video recorded automatically, and the total distance of horizontal movement was calculated in a 3-minute time frame. The test was carried out at night under the special light for OFT with all the other laboratory lights off. The box was cleaned up with 75% ethanol between each test.

#### Mechanical hyperalgesia (von Frey test)

The mechanical hyperalgesia was determined via assessing paw withdrawal threshold (PWT) to mechanical stimuli using a series of von Frey filaments (Stoelting Co., Wood Dale, IL, USA). The rat was placed individually in a transparent plastic box (28 × 25 × 21 cm) with a metal wire mesh floor that allowed full access to paws from beneath and adapted to the testing environment for at least 20 min. Ten von Frey filaments with approximately equal logarithmic incremental (0.17) bending forces were selected (von Frey numbers: 3.61, 3.84, 4.08, 4.17, 4.31, 4.56, 4.74, 4.93, 5.07, and 5.18, equivalent to: 0.4, 0.6, 1.0, 1.4, 2.0, 4.0, 6.0, 8.0, 10.0, and 15.0 g, respectively). The test was initiated with the filament 4.31, in the middle of the series. Whenever a positive or negative response to a given filament occurred, the next smaller or higher filament was applied. A positive response was recorded as the rat’s paw withdrew rapidly when a filament was added or removed. Pattern of positive and negative responses were recorded and converted into a 50% threshold using the provided formula [21].

#### Thermal hyperalgesia (Hargreaves test)

Thermal analgesia was measured using a hot plate machine (YSL-6B, Shanghai Precision Instrument Co., Ltd., China), and the protocol was the same as we reported in a previous publication [22]. Before the thermal sensitivity test, rats were placed on a temperature-controlled glass plate (32 °C) within a Plexiglass compartment and allowed to acclimate for at least 1 h. A light source (50 °C) was then placed under the hind-paw. The latency of paw withdrawal (LPW), a behavioral measure of thermal sensitization, from the light source was evaluated. Each foot was tested fivefold at 3-minute intervals to avoid peripheral sensitization effects.

#### Forced swimming test (FST)

The FST test is slightly different from the method created by Porsolt et al. [23]. We used a single-day procedure according to the actual situation. The rats were placed separately in a transparent glass tank with a water temperature of 25 ± 2 °C for the FST, and the accumulated immobility time of the rats in the water was recorded for 5 min. The rat immobility in water was defined as rat body huddle up, the forepaws stop moving, the hind paws occasional moving but held in a vertical position, and the nostrils were above the water surface. The water was changed between each test.

### High performance liquid chromatography (HPLC)

HPLC technology was applied using ESA 5600A Coularray Detector-8 and associated equipment (ESA Inc., MA, USA) to measure the concentrations of monoamine neurotransmitters serotonin (5-HT), norepinephrine (NE), dopamine (DA), and their metabolic products 5-hydroxyindoleacetic acid (5-HIAA), Homovanillic acid (HVA), 3,4-Dihydroxyphenylacetic acid (DOPAC) in raphe nucleus region, respectively. The experimental conditions were set as follows: pH: 3.0, flow rate: 0.6 ml/min, sample injection volume: 20 μl, column temperature: 30 °C. Three electrical potentials were set for the experiments as − 50, 150, and 350 mV. All reagents were purchased from Sigma-Aldrich (St. Louis, MO, USA). The results were analyzed using ESA software work station, and the concentration (neurotransmitter/tissue, ng/mg) of each sample was calculated.

### Enzyme-linked immunosorbent assay

Serum levels of HPA-axis hormones ACTH, CORT, and inflammatory cytokines IL-1β, IL-6, TNF-α, IFN-γ, IL-4, IL-10 were detected using enzyme linked immunosorbent assay kits (Abcam Inc., ON, Canada; ab263880, ab285260, ab255730, ab234570, ab236712, ab239425, ab100770, ab214566, respectively) in duplicate according to the manufacturer’s instructions.

### Statistical analysis

Statistical analysis was performed using SPSS Statistics (IBM SPSS Statistics for Windows, Version 22.0. Armonk, NY, USA) and Prism 9.0 software (GraphPad Software, San Diego, CA, USA). Values were expressed as the mean ± standard deviation (SD). Differences between two groups were compared by *t* test. Correlation analysis was performed using Spearman’s rank analysis. The *p* value < 0.05 was considered significant.

## Results

### Body weight and weight gain

**Fig. 2:**
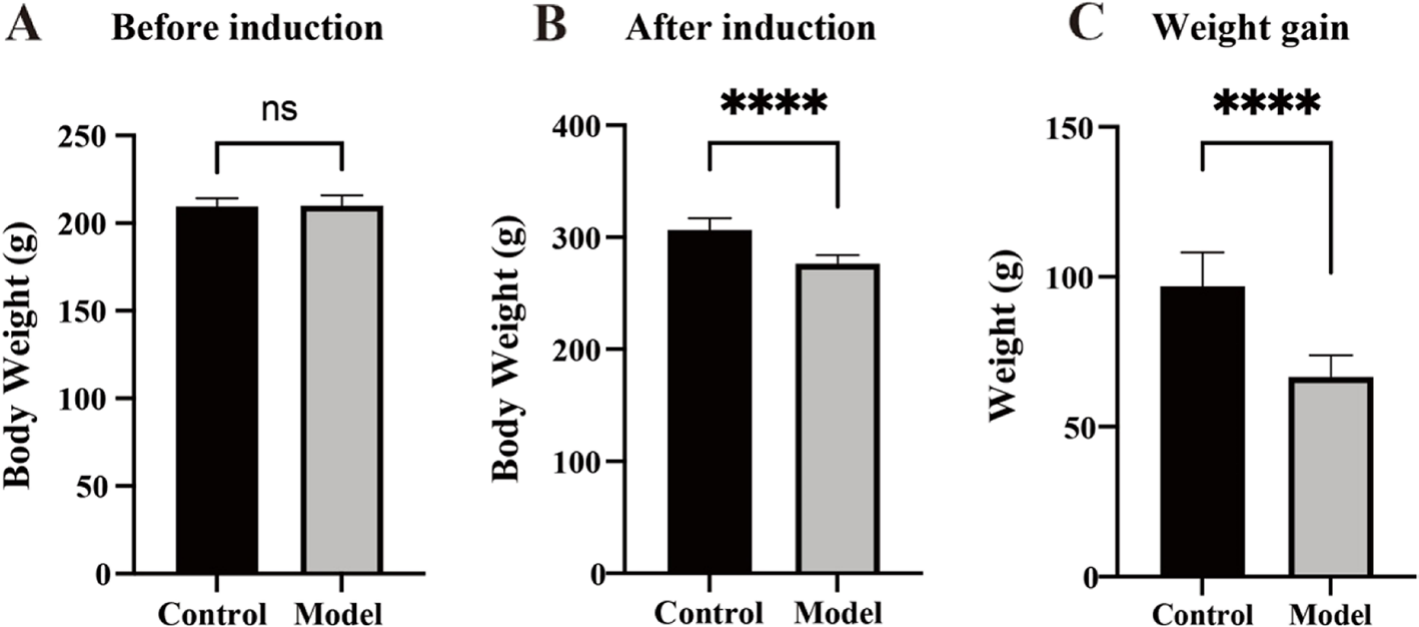
Effects of reserpine injection on body weight. **A** No significant difference existed in body weight between the two groups before reserpine injection. **B** The model group showed lower body weight after reserpine injection. **C** The model group showed decreased body weight gain. Data is expressed as the mean ± SD. ****p < 0.0001, compared with their respective control group

There was no significant difference in body weight between the control and model groups before reserpine injection (*t* = 0.2184, *p* = 0.8296, *n* = 10) (Fig. 2A). After 14 days of continuous reserpine injection, the body weight of rats in the model group was significantly lower than that in the control group (*t* = 7.407, *p* < 0.0001, *n* = 10) (Fig. 2B), and a significant difference in body weight gain was observed between the two groups (*t* = 7.237, *p* < 0.0001, *n* = 10) (Fig. 2C).

### Reserpine-induced changes in depressive behaviors and comorbid hyperalgesia

**Fig. 3:**
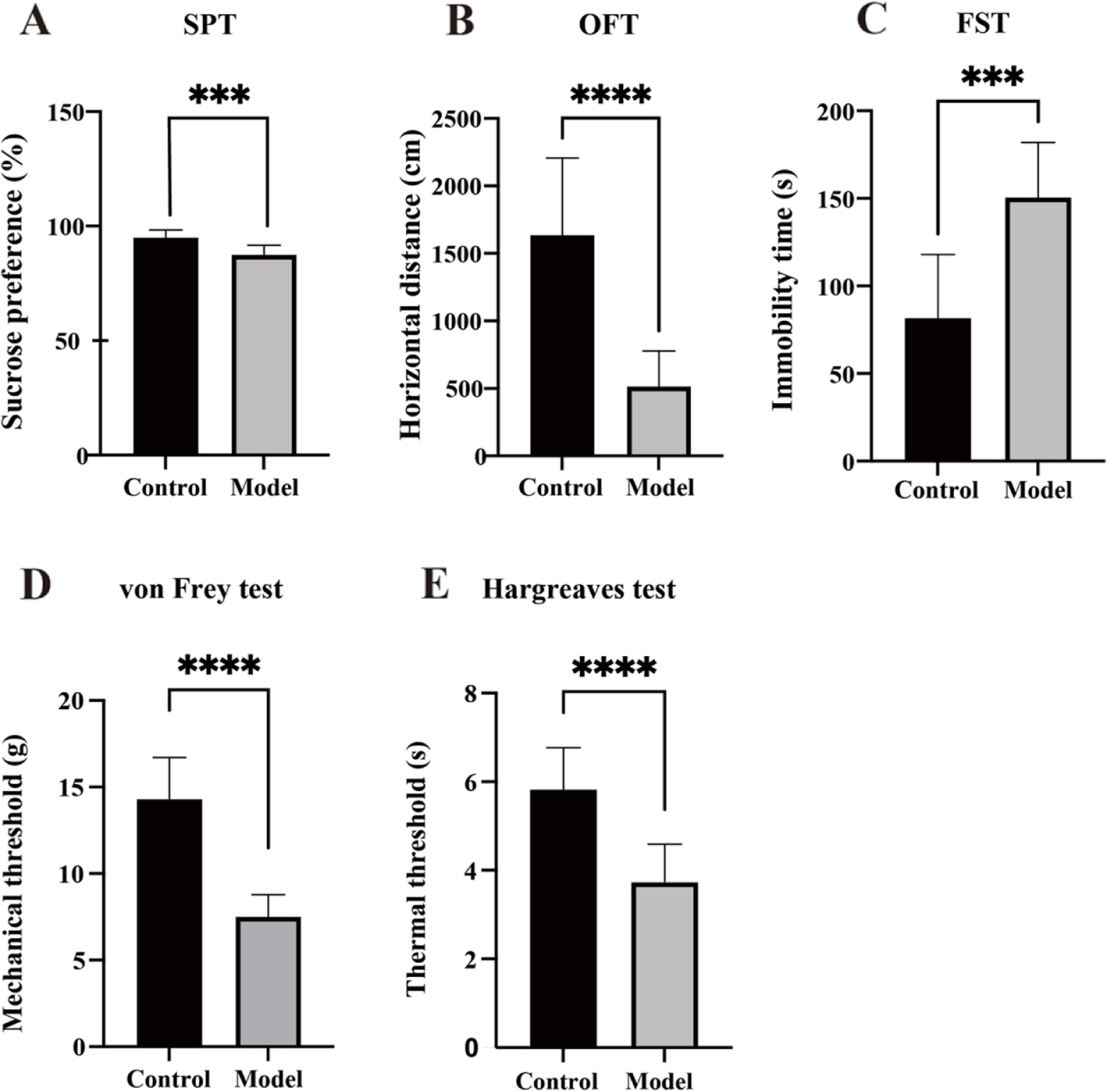
Reserpine-induced depressive behaviors and hyperalgesia. **A** The model group showed decreased sucrose preference. **B** The model group showed decreased horizontal movement distance in the OFT. **C** The model group showed longer immobility time in the FST. **D** The model group showed decreased PWT in the von Frey test. **E** The model group showed decreased thermal threshold in the Hargreaves test. Data is expressed as the mean ± SD. ***p < 0.005, ****p < 0.0001, compared with their respective control group

Following reserpine injection, rats in the model group showed significantly decreased sucrose preference (*t* = 4.488, *p* = 0.0003, *n* = 10) (Fig. 3A) and horizontal movement distance (*t* = 5.643, *p* < 0.0001, *n* = 10) (Fig. 3B), and increased immobility time in the FST (*t* = 4.535, *p* = 0.0003, *n* = 10) (Fig. 3C). Moreover, a significant decrease in PWT (*t* = 7.905, *p* < 0.0001, *n* = 10) (Fig. 3D) and LPW (*t* = 5.218, *p* < 0.0001, *n* = 10) (Fig. 3E) was observed using the von Frey test and a hot stimulus, respectively.

### Reserpine-induced changes in monoamine neurotransmitters and their metabolic products in raphe nucleus

**Fig. 4:**
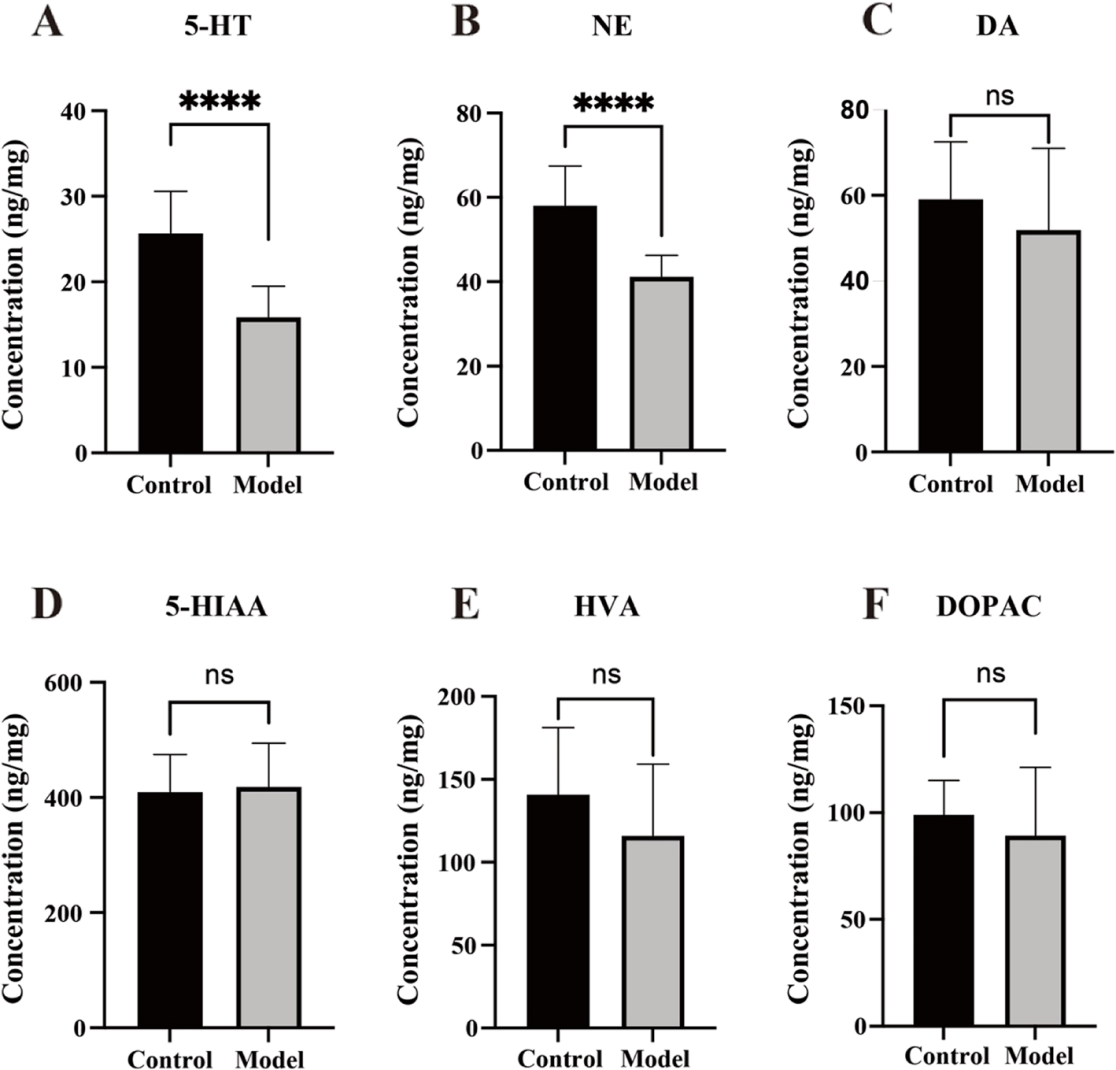
Reserpine-induced changes in monoamine neurotransmitters and their metabolic products. **A** The model group showed decreased concentration of 5-HT. **B** The model group showed decreased concentration of NE. **C** No significant difference existed in concentration of DA. **D** No significant difference existed in concentration of 5-HIAA. **E** No significant difference existed in concentration of HVA. **F** No significant difference existed in concentration of DOPAC. Data is expressed as the mean ± SD. ****p < 0.0001, compared with their respective control group

The concentrations of 5-HT (*t* = 5.111, *p* < 0.0001, *n* = 10) (Fig. 4A) and NE (*t* = 5.008, *p* < 0.0001, *n* = 10) (Fig. 4B) in the model group decreased after the reserpine injection. No significant difference was detected in DA (*t* = 0.9778, *p* = 0.3411, *n* = 10) (Fig. 4C), 5-HIAA (*t* = 0.2883, *p* = 0.7764, *n* = 10) (Fig. 4D), HVA (*t* = 1.334, *p* = 0.1989, *n* = 10) (Fig. 4E), or DOPAC (*t* = 0.8617, *p* = 0.4002, *n* = 10) (Fig. 4F) levels.

### Reserpine-induced changes in serum levels of HPA-axis hormones

**Fig. 5:**
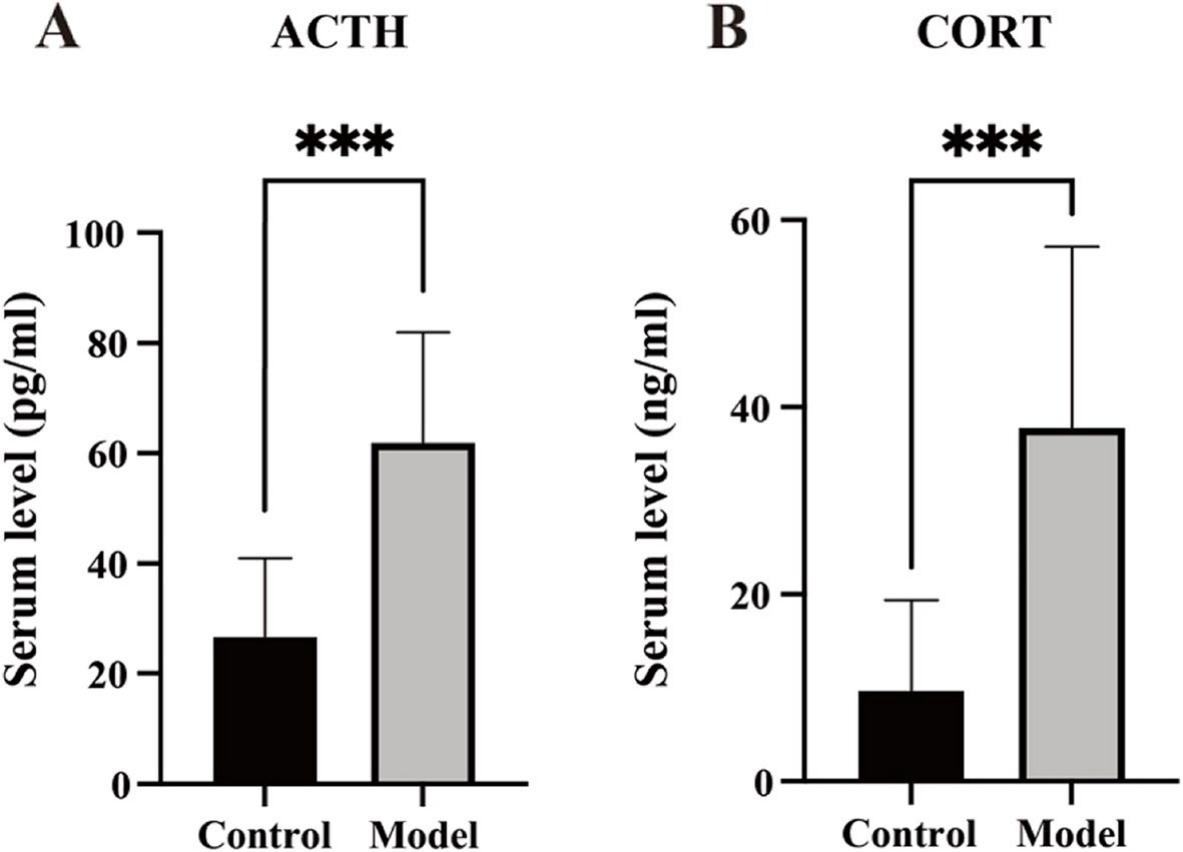
Reserpine-induced changes in serum levels of HPA-axis hormones. **A** The model group showed increased ACTH level. **B** The model group showed increased CORT level. Data is expressed as the mean ± SD. ***p < 0.005, compared with their respective control group

The serum levels of ACTH (*t* = 4.530, *p* = 0.0003, *n* = 10) (Fig. 5A) and CORT (*t* = 4.129, *p* < 0.0006, *n* = 10) (Fig. 5B) in the model group increased after the reserpine injection.

### Reserpine-induced changes in serum cytokine levels

**Fig. 6:**
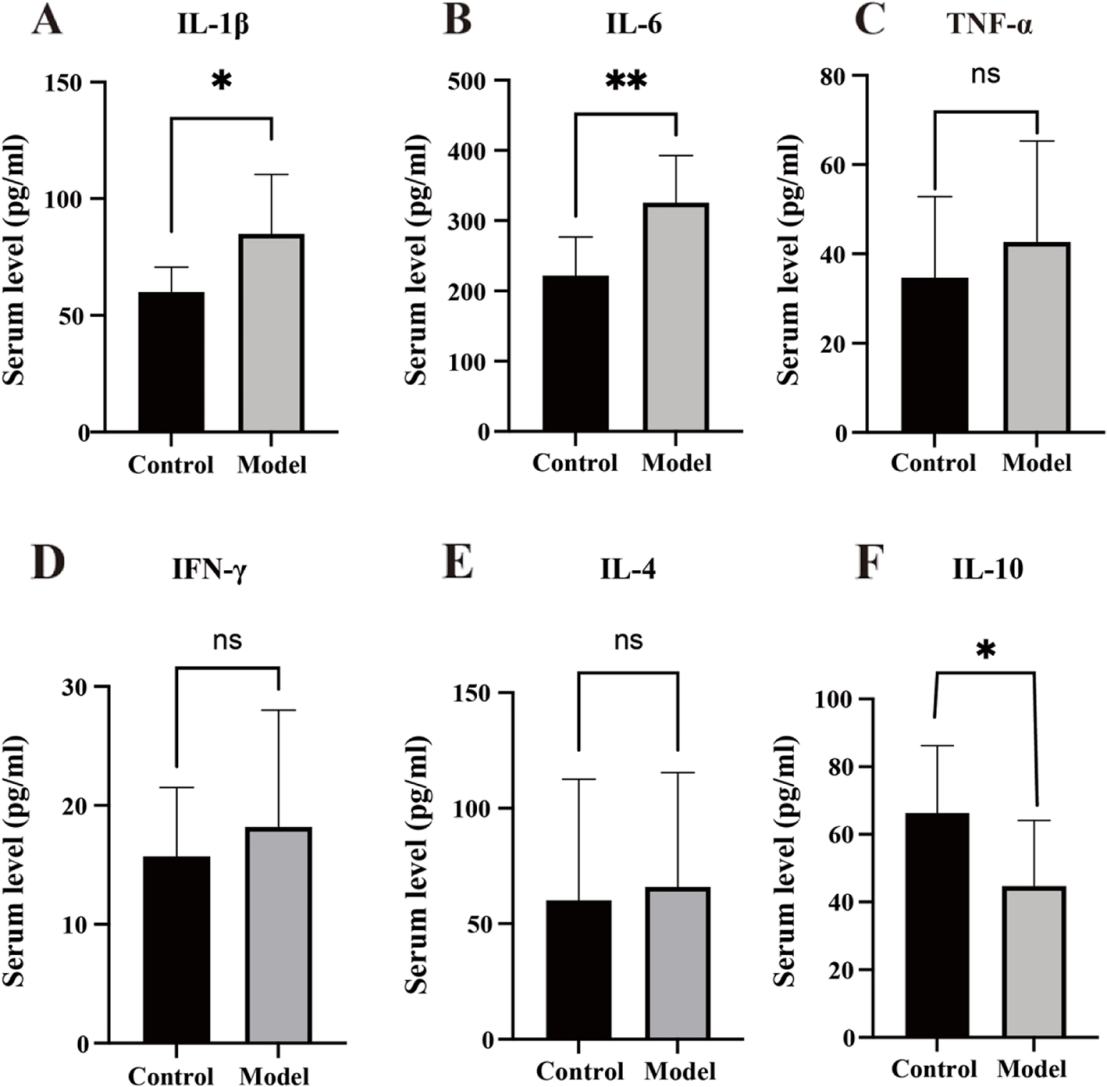
Reserpine-induced changes in serum cytokines. **A** The model group showed increased IL-1β level. **B** The model group showed increased IL-6 level. **C** No significant difference existed in concentration of TNF-α level. **D** No significant difference existed in concentration of IFN-γ level. **E** No significant difference existed in concentration of IL-4 level. **F** The model group showed decreased IL-10 level. Data is expressed as the mean ± SD. *p < 0.05, **p < 0.001, compared with their respective control group

The serum levels of IL-1β (*t* = 2.839, *p* = 0.0109, *n* = 10) (Fig. 6A) and IL-6 (*t* = 3.805, *p* = 0.0013, *n* = 10) (Fig. 6B) in the model group increased after the reserpine injection, while the IL-10 level decreased (*t* = 2.454, *p* = 0.0245, *n* = 10) (Fig. 6F). No significant difference was detected in TNF-α (*t* = 0.8747, *p* = 0.3932, *n* = 10) (Fig. 6C), IFN-γ (*t* = 0.6846, *p* = 0.5023, *n* = 10) (Fig. 6D), or IL-4 (*t* = 0.2573, *p* = 0.7999, *n* = 10) (Fig. 6E) levels.

### Correlation between depression-related and pain-related behaviors

**Fig. 7:**
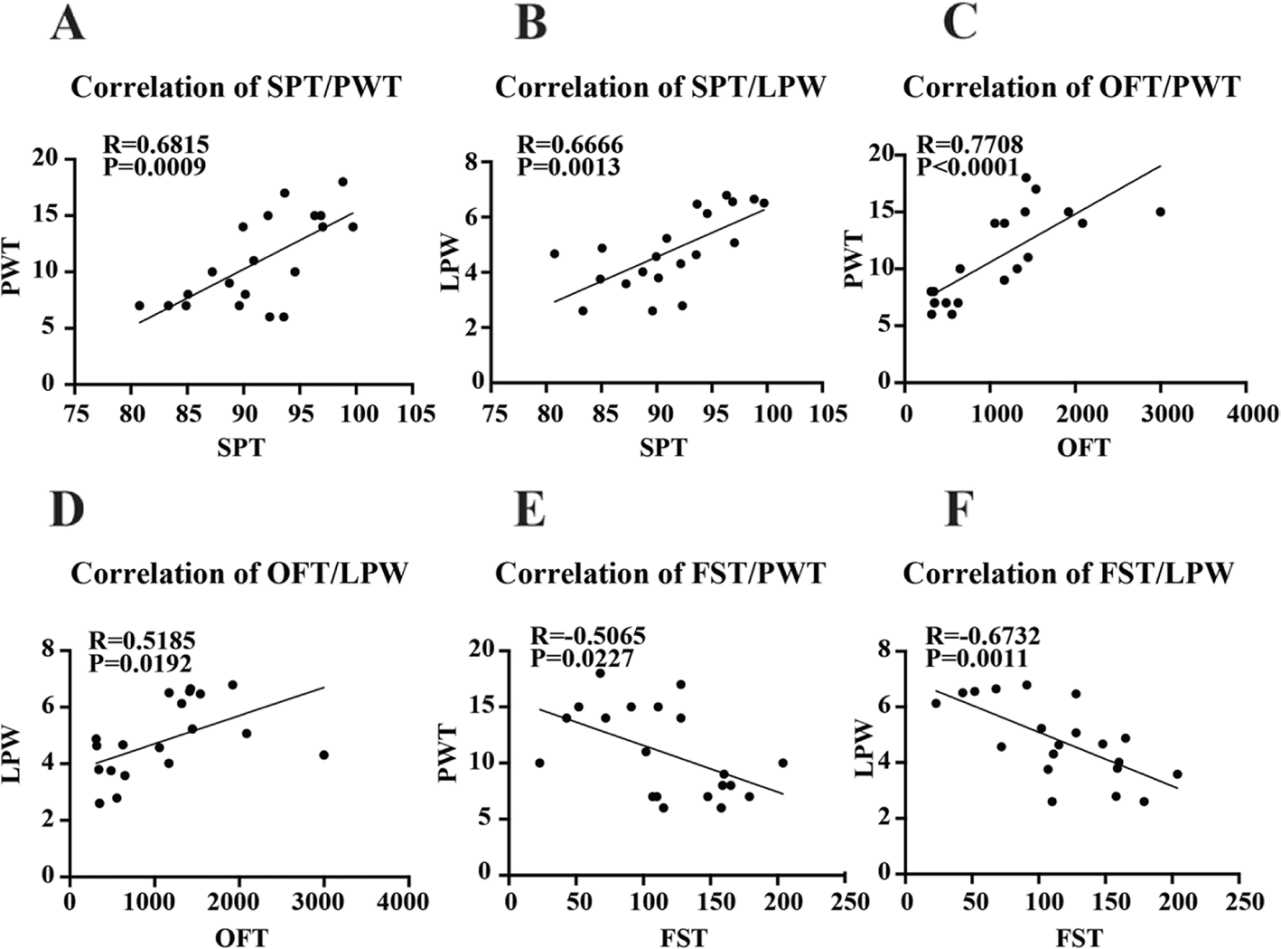
Correlation of reserpine-induced depression-related and pain-related behaviors. **A** The correlation between SPT and PWT. **B** The correlation between SPT and LPW. **C** The correlation between OFT and PWT. **D** The correlation between OFT and LPW. **E** The correlation between FST and PWT. **F** The correlation between FST and LPW. Data is expressed as the mean ± SD

The results of the correlation analysis between depression-related behaviors and pain-related behaviors are shown in Fig. 7. SPT, OFT and FST were linearly correlated with PWT and LPW, respectively, and the differences were statistically significant.

## Discussion

The association between pain and depression has been known since 1997 [24]. Based on the fact that pain and depression are frequent co-morbid disorders [2, 6] and that some antidepressants are effective for pain [25, 26], the association of chronic pain with depression is becoming increasingly recognized. In the present study, we examined some common pathways of pain and depression comorbidity based on a reserpine-induced rat model. Compared with the control group, rats in the model group showed a lower sucrose preference, reflecting anhedonia, indicating a core symptom of depression. PWT, reflecting mechanical hyperalgesia, and LPW, reflecting thermal hyperalgesia, were decreased in the model group, indicating the phenotype of pain. Considering that the FST can influence pain sensitivity in rodents [27], we finished the von Frey test and the Hargreaves test before the FST. The results of all of the behavioral tests further confirmed the reliability of the modeling method of reserpine-induced hyperalgesia and depression comorbidity. The linear correlation between depression-related behavior and pain-related behavior further confirmed the co-existence of pain and depression.

Monoamine neurotransmitters including 5-HT [28], NE [29], and DA [30] are among the most studied candidates in both the field of chronic pain and depression. Studies have shown that 5-HT has a key role in the pathogenesis and pharmacotherapy of depression [31] and in pain transmission [32]. NE plays an important role in regulating pain sensitivity [33], and NE deficiency is also a risk factor for depression [34]. Dopamine dysfunction is associated with depression [35] and increased pain sensitivity in several chronic pain conditions [36, 37]. Clinical practice has shown that some selective serotonin reuptake inhibitors (SSRIs) [38], serotonin norepinephrine reuptake inhibitors (SNRI) [29], and norepinephrine reuptake inhibitors (NRIs) [39] showed good efficacy in the control of both depression and pain. Raphe nucleus is abundant of serotonergic neurons [40]. The present study found that the level of 5-HT and NE in the raphe nucleus in the model group was lower than the control group, and the result was consistent with the previous research [41].

The second facet of the pain-depression comorbidity is the involvement of HPA axis [42]. Patients with depression often have elevated baseline CORT levels compared to healthy controls [43, 44]. There are few studies on the relationship between pain and the HPA axis, and the conclusions are not always consistent. It has been reported that patients with temporomandibular disorders with pain as one of the main symptoms have hyperfunction of HPA axis [45]. Whereas, in another study in white (non-black) women experiencing chronic pelvic pain, a blunted response of HPA axis was related to pain severity was observed [46]. The results of our present study showed that there was hyperfunction of HPA axis in rats with pain and depression comorbidity, as both ACTH and CORT in the model group were higher than those in control. More in-depth studies are needed on the interaction and mechanism between pain and depression comorbidity and HPA axis.

Over the past decade, numerous studies have demonstrated that cytokines seem to play an important role in both pain [47] and depression [48]. Elevated IL-1β [49], IL-6 [49, 50], TNF-α [49] and IL-17 [50] levels were observed in clinical patients with pain and depression comorbidity. Pre-clinical studies focusing on different chronic pain models with comorbid depression also reported upregulation of IL-1β [51], IL-6 [52, 53] and TNF-α [52, 53]. We observed elevated IL-1β, IL-6 and reduced IL-10 in the reserpine-induced hyperalgesia and depression comorbidity model group in our present study. Previous studies have reported reduced IL-10 in patients with depression [54] and pain [55], respectively, but our study is the first to report reduced IL-10 in depression and pain comorbidity models. We hypothesized that there may be a low Th2 or M2 immune response in pain depression comorbidities since IL-10 is a Th2 or M2-associated cytokine [56]. This also provides some hints for our future research direction, and may become a target for the treatment of pain and depression comorbidity.

Although our study comprehensively investigated alterations in monoamine neurotransmitters, HPA axis, and immune inflammation in hyperalgesia and depression comorbidity, there are still some limitations. First, our study is based on a reserpine-induced rat model, and it is uncertain that the same changes are present in clinical patients. Secondly, our study just clarified the phenomena, and more and more in-depth mechanism studies are needed to clarify the causes of these alterations.

## Conclusions

In summary, our study is the first to comprehensively detect alterations in monoamine neurotransmitters, HPA axis, and immune inflammation based on a reserpine-induced rat model. We confirmed that repeated reserpine injection induced depressive behaviors and pain phenotypes, and increased 5-HT, NE, ACTH, and CORT were detected in the model group. Elevated IL-1β, IL-6 and lowered IL-10 in the model rats indicated there may be a low Th2 or/and M2 immune response in pain and depression comorbidities. More research in these areas is needed to understand the pathogenesis of the disease, so as to find more and better therapeutic targets.

## Data Availability

The datasets used and/or analyzed during the current study are available from the corresponding author on reasonable request.

## References

[1] Smith KA, Alt JA (2020). The relationship of chronic rhinosinusitis and depression. Curr Opin Otolaryngol Head Neck Surg.

[2] Zis P, Daskalaki A, Bountouni I, Sykioti P, Varrassi G, Paladini A (2017). Depression and chronic pain in the elderly: links and management challenges. Clin Interv Aging.

[3] Hassett AL, Marshall E, Bailey AM, Moser S, Clauw DJ, Hooten WM, Urquhart A, Brummett CM (2018). Changes in anxiety and depression are mediated by changes in pain severity in patients undergoing lower-extremity Total joint Arthroplasty. Reg Anesth Pain Med.

[4] Jackson T, Chen H, Iezzi T, Yee M, Chen F (2014). Prevalence and correlates of chronic pain in a random population study of adults in Chongqing, China. Clin J Pain.

[5] Cabrera-León A, Cantero-Braojos M, Garcia-Fernandez L (2018). Guerra de Hoyos JA: living with disabling chronic pain: results from a face-to-face cross-sectional population-based study. BMJ Open.

[6] Roughan WH, Campos AI, García-Marín LM, Cuéllar-Partida G, Lupton MK, Hickie IB, Medland SE, Wray NR, Byrne EM, Ngo TT (2021). Comorbid chronic pain and depression: shared risk factors and differential antidepressant effectiveness. Front Psychiatry.

[7] Wang XQ, Peng MS, Weng LM, Zheng YL, Zhang ZJ, Chen PJ (2019). Bibliometric study of the comorbidity of pain and depression research. Neural Plast.

[8] Russell IJ, Vaeroy H, Javors M, Nyberg F (1992). Cerebrospinal fluid biogenic amine metabolites in fibromyalgia/fibrositis syndrome and rheumatoid arthritis. Arthritis Rheum.

[9] Mann JJ, Malone KM (1997). Cerebrospinal fluid amines and higher-lethality suicide attempts in depressed inpatients. Biol Psychiatry.

[10] Wingenfeld K, Heim C, Schmidt I, Wagner D, Meinlschmidt G, Hellhammer DH (2008). HPA axis reactivity and lymphocyte glucocorticoid sensitivity in fibromyalgia syndrome and chronic pelvic pain. Psychosom Med.

[11] Dalm S, Karssen AM, Meijer OC, Belanoff JK, de Kloet ER (2019). Resetting the stress system with a mifepristone challenge. Cell Mol Neurobiol.

[12] Clark AK, Old EA, Malcangio M (2013). Neuropathic pain and cytokines: current perspectives. J Pain Res.

[13] Capuron L, Miller AH (2011). Immune system to brain signaling: neuropsychopharmacological implications. Pharmacol Ther.

[14] Kremer M, Becker LJ, Barrot M, Yalcin I (2021). How to study anxiety and depression in rodent models of chronic pain?. Eur J Neurosci.

[15] Alba-Delgado C, Llorca-Torralba M, Horrillo I, Ortega JE, Mico JA, Sánchez-Blázquez P, Meana JJ, Berrocoso E (2013). Chronic pain leads to concomitant noradrenergic impairment and mood disorders. Biol Psychiatry.

[16] Piardi LN, Pagliusi M, Bonet I, Brandão AF, Magalhães SF, Zanelatto FB, Tambeli CH, Parada CA, Sartori CR (2020). Social stress as a trigger for depressive-like behavior and persistent hyperalgesia in mice: study of the comorbidity between depression and chronic pain. J Affect Disord.

[17] Wang N, Shi M, Wang JY, Luo F (2013). Brain-network mechanisms underlying the divergent effects of depression on spontaneous versus evoked pain in rats: a multiple single-unit study. Exp Neurol.

[18] Stevenson GW, Mercer H, Cormier J, Dunbar C, Benoit L, Adams C, Jezierski J, Luginbuhl A, Bilsky EJ (2011). Monosodium iodoacetate-induced osteoarthritis produces pain-depressed wheel running in rats: implications for preclinical behavioral assessment of chronic pain. Pharmacol Biochem Behav.

[19] Arora V, Kuhad A, Tiwari V, Chopra K (2011). Curcumin ameliorates reserpine-induced pain-depression dyad: behavioural, biochemical, neurochemical and molecular evidences. Psychoneuroendocrinology.

[20] Shi W, Zhang S, Lu Y, Wang Y, Zhao J, Li L (2022). T cell responses in depressed mice induced by chronic unpredictable mild stress. J Affect Disord.

[21] Chaplan SR, Bach FW, Pogrel JW, Chung JM, Yaksh TL (1994). Quantitative assessment of tactile allodynia in the rat paw. J Neurosci Methods.

[22] Zhao J, Gao X, Wang A, Wang Y, Du Y, Li L, Li M, Li C, Jin X, Zhao M (2019). Depression comorbid with hyperalgesia: different roles of neuroinflammation induced by chronic stress and hypercortisolism. J Affect Disord.

[23] Porsolt RD, Le Pichon M, Jalfre M (1977). Depression: a new animal model sensitive to antidepressant treatments. Nature.

[24] Fishbain DA, Cutler R, Rosomoff HL, Rosomoff RS (1997). Chronic pain-associated depression: antecedent or consequence of chronic pain? A review. Clin J Pain.

[25] Häuser W, Wolfe F, Tölle T, Uçeyler N, Sommer C (2012). The role of antidepressants in the management of fibromyalgia syndrome: a systematic review and meta-analysis. CNS Drugs.

[26] Rosenberg MB, Carroll FI, Negus SS (2013). Effects of monoamine reuptake inhibitors in assays of acute pain-stimulated and pain-depressed behavior in rats. J Pain.

[27] Łapo IB, Konarzewski M, Sadowski B (2003). Analgesia induced by swim stress: interaction between analgesic and thermoregulatory mechanisms. Pflugers Arch.

[28] Zhou W, Jin Y, Meng Q, Zhu X, Bai T, Tian Y, Mao Y, Wang L, Xie W, Zhong H (2019). A neural circuit for comorbid depressive symptoms in chronic pain. Nat Neurosci.

[29] Goldenberg DL, Clauw DJ, Palmer RH, Mease P, Chen W, Gendreau RM (2010). Durability of therapeutic response to milnacipran treatment for fibromyalgia. Results of a randomized, double-blind, monotherapy 6-month extension study. Pain Med.

[30] Serafini RA, Pryce KD, Zachariou V (2020). The mesolimbic dopamine system in chronic pain and associated affective comorbidities. Biol Psychiatry.

[31] Nemeroff CB, Owens MJ (2009). The role of serotonin in the pathophysiology of depression: as important as ever. Clin Chem.

[32] Ossipov MH, Morimura K, Porreca F (2014). Descending pain modulation and chronification of pain. Curr Opin Support Palliat Care.

[33] Stahl S, Briley M (2004). Understanding pain in depression. Hum Psychopharmacol.

[34] Schildkraut JJ (1967). The catecholamine hypothesis of affective disorders. A review of supporting evidence. Int J Psychiatry.

[35] Friedman AK, Walsh JJ, Juarez B, Ku SM, Chaudhury D, Wang J, Li X, Dietz DM, Pan N, Vialou VF (2014). Enhancing depression mechanisms in midbrain dopamine neurons achieves homeostatic resilience. Science.

[36] Barbanti P, Aurilia C, Egeo G, Fofi L, Guadagni F, Ferroni P (2020). Dopaminergic symptoms in migraine: a cross-sectional study on 1148 consecutive headache center-based patients. Cephalalgia.

[37] Vargas-Alarcón G, Fragoso JM, Cruz-Robles D, Vargas A, Vargas A, Lao-Villadóniga JI, García-Fructuoso F, Ramos-Kuri M, Hernández F, Springall R (2007). Catechol-O-methyltransferase gene haplotypes in Mexican and Spanish patients with fibromyalgia. Arthritis Res Ther.

[38] Jaracz J, Gattner K, Jaracz K, Górna K, Moczko J, Hauser J (2018). Is venlafaxine more effective than Escitalopram and Nortriptyline in the Management of Painful Symptoms in patients with major depression?. Pharmacopsychiatry.

[39] Leventhal L, Smith V, Hornby G, Andree TH, Brandt MR, Rogers KE (2007). Differential and synergistic effects of selective norepinephrine and serotonin reuptake inhibitors in rodent models of pain. J Pharmacol Exp Ther.

[40] Hale MW, Dady KF, Evans AK, Lowry CA (2011). Evidence for in vivo thermosensitivity of serotonergic neurons in the rat dorsal raphe nucleus and raphe pallidus nucleus implicated in thermoregulatory cooling. Exp Neurol.

[41] Nagakura Y, Oe T, Aoki T, Matsuoka N (2009). Biogenic amine depletion causes chronic muscular pain and tactile allodynia accompanied by depression: a putative animal model of fibromyalgia. Pain.

[42] Thornton LM, Andersen BL, Blakely WP (2010). The pain, depression, and fatigue symptom cluster in advanced breast cancer: covariation with the hypothalamic-pituitary-adrenal axis and the sympathetic nervous system. Health Psychol.

[43] Contreras F, Menchon JM, Urretavizcaya M, Navarro MA, Vallejo J, Parker G (2007). Hormonal differences between psychotic and non-psychotic melancholic depression. J Affect Disord.

[44] Keller J, Flores B, Gomez RG, Solvason HB, Kenna H, Williams GH, Schatzberg AF (2006). Cortisol circadian rhythm alterations in psychotic major depression. Biol Psychiatry.

[45] Staniszewski K, Lygre H, Bifulco E, Kvinnsland S, Willassen L, Helgeland E, Berge T, Rosén A (2018). Temporomandibular disorders related to stress and HPA-Axis regulation. Pain Res Manag.

[46] Ortiz R, Gemmill JAL, Sinaii N, Stegmann B, Khachikyan I, Chrousos G, Segars J, Stratton P (2020). Hypothalamic-pituitary-adrenal Axis responses in women with endometriosis-related chronic pelvic pain. Reprod Sci.

[47] Ludwig J, Binder A, Steinmann J, Wasner G, Baron R (2008). Cytokine expression in serum and cerebrospinal fluid in non-inflammatory polyneuropathies. J Neurol Neurosurg Psychiatry.

[48] Dowlati Y, Herrmann N, Swardfager W, Liu H, Sham L, Reim EK, Lanctôt KL (2010). A meta-analysis of cytokines in major depression. Biol Psychiatry.

[49] Hu C, Yang H, Zhao Y, Chen X, Dong Y, Li L, Dong Y, Cui J, Zhu T, Zheng P (2016). The role of inflammatory cytokines and ERK1/2 signaling in chronic prostatitis/chronic pelvic pain syndrome with related mental health disorders. Sci Rep.

[50] Li YC, Chou YC, Chen HC, Lu CC, Chang DM (2019). Interleukin-6 and interleukin-17 are related to depression in patients with rheumatoid arthritis. Int J Rheum Dis.

[51] Li L, Zou Y, Liu B, Yang R, Yang J, Sun M, Li Z, Xu X, Li G, Liu S (2020). Contribution of the P2X4 receptor in rat Hippocampus to the comorbidity of chronic pain and depression. ACS Chem Neurosci.

[52] Jiang X, Yan Q, Liu F, Jing C, Ding L, Zhang L, Pang C (2018). Chronic trans-astaxanthin treatment exerts antihyperalgesic effect and corrects co-morbid depressive like behaviors in mice with chronic pain. Neurosci Lett.

[53] Brüning CA, Martini F, Soares SM, Sampaio TB, Gai BM, Duarte MM, Nogueira CW (2015). M-Trifluoromethyl-diphenyl diselenide, a multi-target selenium compound, prevented mechanical allodynia and depressive-like behavior in a mouse comorbid pain and depression model. Prog Neuro-Psychopharmacol Biol Psychiatry.

[54] Zhang HY, Wang Y, He Y, Wang T, Huang XH, Zhao CM, Zhang L, Li SW, Wang C, Qu YN (2020). A1 astrocytes contribute to murine depression-like behavior and cognitive dysfunction, which can be alleviated by IL-10 or fluorocitrate treatment. J Neuroinflammation.

[55] Uçeyler N, Eberle T, Rolke R, Birklein F, Sommer C (2007). Differential expression patterns of cytokines in complex regional pain syndrome. Pain.

[56] Mantovani A, Sica A, Sozzani S, Allavena P, Vecchi A, Locati M (2004). The chemokine system in diverse forms of macrophage activation and polarization. Trends Immunol.

